# Multiple synchronous primary melanomas; A case report

**DOI:** 10.1016/j.jpra.2025.09.001

**Published:** 2025-09-09

**Authors:** Nikolaj Warming, Erik Gadsbøll, Nulvin Djebbara-Bozo, Anders Munk, Helle Skyum

**Affiliations:** a Department of Dermatology and Venereology, Aalborg University Hospital, Mølleparkvej 10, Aalborg, Nordjylland, Denmark; b Department of Plastic Surgery, Aalborg University Hospital, Søndre Skovvej 3, Aalborg, Nordjylland, Denmark

**Keywords:** Melanoma, Skin cancer, Multiple primary melanomas, Synchronous multiple primary melanoma

## Abstract

Cutaneous melanoma is a highly aggressive form of skin cancer, responsible for >90 % of deaths related to skin tumors, despite accounting for only a small proportion of total cutaneous tumors.

Cutaneous melanoma is the fifth most commonly diagnosed cancer in both females and males and there is an annual increase of around 3–7 %.

In recent years, survival rates have improved due to health education campaigns, prevention strategies and the development of new treatments for advanced disease.

Melanoma patients have an increased risk of developing a new melanoma, although the risk is still low. Most new melanomas are diagnosed within 5 years of the first melanoma. A small fraction of patients are diagnosed with more than one melanoma within 3 months of the first melanoma, referred to as synchronous melanomas. As this is a rare phenomenon, the available literature remains limited. Some studies have shown improved survival in this group and that the subsequent melanoma was significantly thinner; however further research is needed to fully understand this phenomenon.

Here we present a case with a patient with six synchronous melanomas diagnosed within 19 days. The patient underwent genetic screening but no pathogenic gene variants were found.

This case highlights the importance of thorough skin examination at the time of melanoma diagnosis.

To our knowledge, this is the first reported case of six synchronous melanomas in the absence of identifiable genetic predisposition. The case demonstrates the need for further research.

## Background

Cutaneous melanoma is a highly aggressive form of skin cancer, which is responsible for over 90 % of deaths related to this specific group of tumors.^1^, ^2^, ^3^ However, cutaneous melanoma only represents a small part of the total cutaneous tumors.^2^

The annual increase in cutaneous melanomas is estimated to be between 3–7 %, and it is the fifth most commonly diagnosed cancer among both females and males.^2^^,^^4^

Due to health education campaigns, prevention strategies and the development of new treatment for disseminated stages, the survival rate for cutaneous melanoma has increased over recent years.^2^

Patients diagnosed with cutaneous melanoma are, from the time of the diagnosis, at increased risk of developing a subsequent malignancy and especially a new primary melanoma (0.2–8.6 %).^2^^,^^5^

The development of more than one primary melanoma in a single patient was described by Pack et al. in 1952 with an incidence of 1,3 % (16 of 1250) and the phenomenon was referred to as multiple primary melanomas (MPM).^2^^,^^3^^,^^7^^,^^8^

The majority of MPM is diagnosed within the first 5 years, while it is also seen later than 10 years after the primary diagnosis.^5^ One case report describes a second primary melanoma 41 years after the diagnosis.^2^

In recent years, subsequent melanomas have been categorized as asynchronous or synchronous in relation to the time of diagnosis.^3^^,^^6^).

Synchronous melanomas (SM) are not clearly defined or standardized in the literature, but are referred to as primary melanomas occurring 1 to 3 months after primary excision of the first melanoma.^1^^,^^3^^,^^6^^,^^8^

According to the literature, 0.5 % of all melanoma patients are diagnosed SM and 26–40 % of patients with MPM are diagnosed SM.^4^^,^^6^^,^^8^

## Case presentation

**Figure 1–2:**
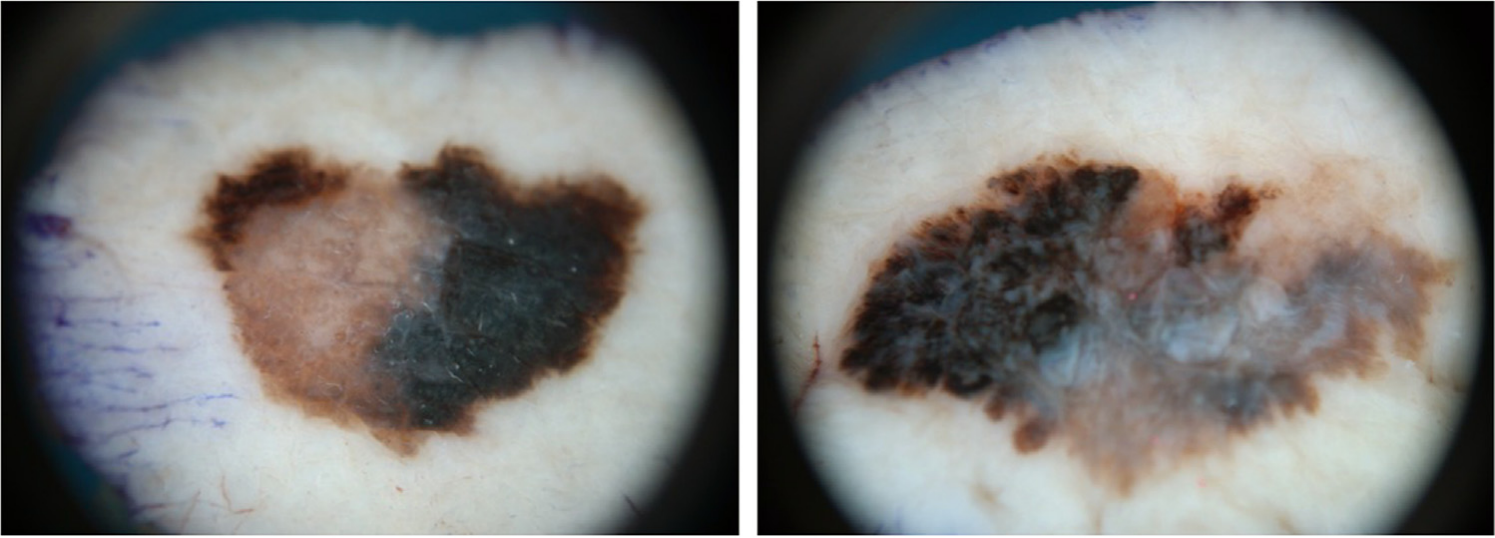
The first and second melanoma magnified in a microscope. T1a-melanoma to the left, T2a-melanoma to the right.

A 71-year-old male patient with a complex medical history, including diabetes, hypertension, hyperlipidemia, chronic kidney disease, and a mechanical aortic valve, was referred to the department of plastic surgery at Aalborg University hospital for evaluation of two suspicious nevi located on the upper back and on the inside of his left knee. No family history of malignant melanoma (MM) was reported, but the patient had a history of regular sun exposure and episodes of severe sun burns particularly during childhood. The nevi were excised according to guidelines of the Danish Melanoma Group (DMG). Pathological examinations concluded two superficial spreading melanomas, microstage pT2a and pT1a Figure 1–2.

**Figure 3–4:**
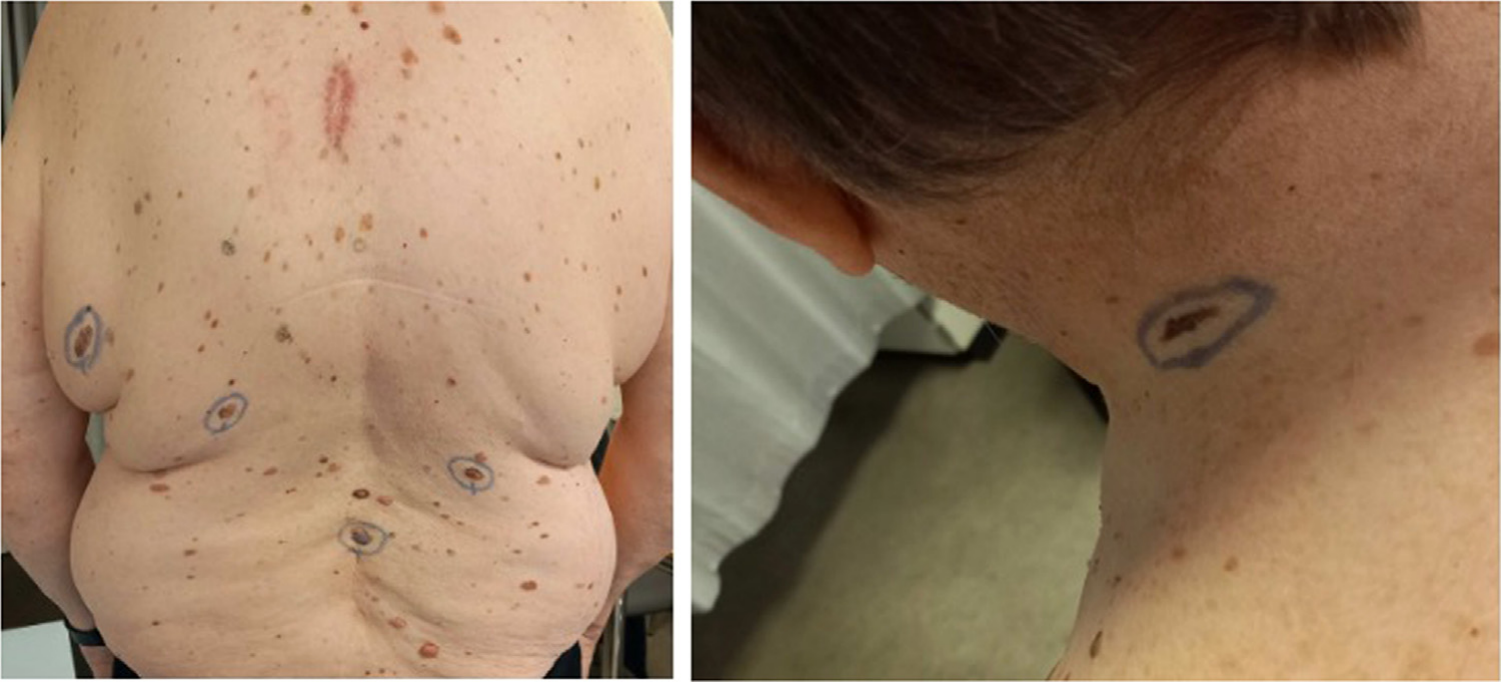
Highlight of the five clinically suspicious nevi (four on the back, one on the neck) found during total skin examination.

The patient underwent a total skin examination (TSE), and palpation of lymph nodes was performed. Further five suspicious naevi were found, four at the back and one on the left side of the neck (Figures 3–4).

**Figure 5–8:**
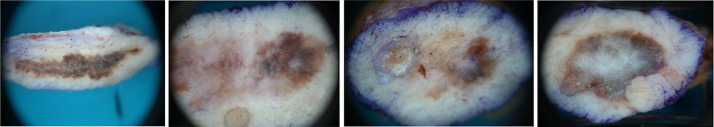
The four subsequent excised melanoma magnified in a microscope (from left to right pTis-, pT1a-, pT1a- and pT2a-melanoma).

All five nevi were excised as a sentinel node (SN) procedure was performed in general anesthesia. Four out of five lesions were microscopically confirmed as new primary superficial spreading melanomas, microstage pT2a and two times pT1a and pTis (Figs. 5–8). As a result of the pathological examination, another SN procedure was performed preceded by a fluorodeoxyglucose (FDG)-positron emission tomography (PET) scan. Neither FDG-PET scan nor the sentinel nodes showed sign of metastasis.

To sum up the total amount of melanomas, the patient had five invasive melanoma (two pT2a and three pT1a) and one “in situ” melanoma excised

Furthermore, the patient volunteered to genetic screening with a hereditary melanoma gene-panel to exclude any disease-predisposing gene variants. The genetic test concluded that no pathogenic gene variants were detected.

The patient was then referred to the department of dermatology, where an automated total body imaging with integrated body mapping system was used to point out other suspicious naevi. Four lesions were excised, all of them microscopically confirmed as benign lesions. A written consent from the patient was collected prior to the case report.

## Discussion

The patient in this case was referred for excision of two nevi suspicious for MM. Nineteen days later four more melanomas were excised bringing the total to six synchronous primary melanomas.

The current diagnosis of SM is inconsistently described with the use of heterogeneous terminology.^3^^,^^7^^,^^8^ The majority of studies regarding SM define it as a second melanoma detected within one months after the primary diagnosis of melanoma.^3^ Other studies define SM as a subsequent melanoma within two to three months after the primary diagnosis.^2^, ^3^, ^4^^,^^6^^,^^8^

Many different factors have been described which potentially contribute to the development of more than one primary cutaneous melanoma. In particular the following factors such as age, outdoor work (>10 years), the number of nevi, fair hair, skin type I-II, recurrent sunburns, familial predisposition to melanoma or dysplastic nevi.^2^^,^^5^ SM have been particularly associated with older male patients, significant sun-exposure and nevus spilus.^3^^,^^6^^,^^8^

The six melanomas in our case were located in three different anatomical regions (one on the left leg, four on the back and one at the neck). Regarding body concordance of a subsequent melanoma, some studies show contradictory results.^3^

A recent retrospective study compared an asynchronous group with a synchronous group. They found a significant higher concordance between anatomical location MPM, when comparing asynchronous and synchronous (38.2 % vs 55.7 %).^8^ Previous studies reported highest correlation for head/neck (80 %), extremities (60 %) and the back (47.5 %).^3^ However, a case report presented three synchronous melanomas located at different anatomic areas.^9^

Several studies have compared pathological characteristics between the first and second primary melanoma regarding MPM patients. They found that the second melanoma was thinner and less frequently ulcerated than the first melanoma.^1^^,^^5^^,^^8^ However, this is not applicable in every case as 13 % of the cases had melanoma with higher tumour thickness.^5^ Sarver et al.^8^ found that the melanoma within patients with a single primary melanoma were significantly thicker than melanomas of patients with MPM, both synchronous and asynchronous. The two groups of MPM also had a significantly thinner secondary melanoma.^8^ Furthermore, SM patients have reportedly increased risk of developing a new primary melanoma (25.7 %) which also tend to develop earlier (Median time 2.9 vs 4.1 years), compared to patient with a single melanoma (8,6 %).^4^ The European consensus-based interdisciplinary guidelines for melanoma recommend the use of dermoscopy for all skin lesions, rather than solely for those that are clinically suspicious.^1^ This approach may lead to the diagnosis of additional melanomas and facilitate synchronous treatment, thereby reducing risk for patients and proving cost-effective for the healthcare system.

### Survival/prognosis

The literature has been ambiguous regarding outcomes and survival when comparing MPM to patient with a single primary melanoma.^3^, ^4^^,^^8^ In recent years, studies have revealed an equal or enhanced survival in MPMs.^4^ A single study used SEER’s and showed a worse prognosis for patients with SM compared to those with a single primary melanoma.^5^^,^^10^

However, a recent study by Sarver et al. showed significantly improved survival probabilities when comparing SM to single melanoma patients.^8^ No difference between patients with asynchronous and synchronous second melanoma was found.^8^ One hypothesis for the enhanced survival is the “immunization effect,” an immune response to melanoma antigens, which slows the subsequent melanoma progression.^4^^,^^8^ Other studies show higher rates of self-reporting, skin self-evaluation, adherence with follow-up programs, and usage of sunscreen/sun protective measures when comparing MPM to patients with a single melanoma.^8^

## Funding

No external funding.

## Declaration of generative AI and AI-assisted technologies in the writing process

During the preparation of this work the authors did not use generative AI and AI-assisted technologies in the writing process.

## Declaration of competing interest

The authors have no conflicts of interest to disclose.
